# Building a symbolic computer algebra toolbox to compute 2D Fourier transforms in polar coordinates

**DOI:** 10.1016/j.mex.2015.03.008

**Published:** 2015-04-01

**Authors:** Edem Dovlo, Natalie Baddour

**Affiliations:** a Centre for Advanced Diffusion-Wave Technologies (CADIFT), Department of Mechanical and Industrial Engineering, University of Toronto, 5 King’s College Rd., Toronto, ON M5S 3G8, Canada; b Department of Mechanical Engineering, University of Ottawa, 161 Louis Pasteur, Ottawa, ON K1N 6N5, Canada

**Keywords:** Computation of 2D Fourier transforms in polar coordinates via symbolic computer algebra (Maple), 2D Fourier transform, Polar coordinates, Symbolic computer algebra, Convolution

## Abstract

The development of a symbolic computer algebra toolbox for the computation of two dimensional (2D) Fourier transforms in polar coordinates is presented. Multidimensional Fourier transforms are widely used in image processing, tomographic reconstructions and in fact any application that requires a multidimensional convolution. By examining a function in the frequency domain, additional information and insights may be obtained. The advantages of our method include:

• The implementation of the 2D Fourier transform in polar coordinates within the toolbox via the combination of two significantly simpler transforms.
• The modular approach along with the idea of *lookup* tables implemented help avoid the issue of indeterminate results which may occur when attempting to directly evaluate the transform.
• The concept also helps prevent unnecessary computation of already known transforms thereby saving memory and processing time.

## Graphical abstract

**Figure:**
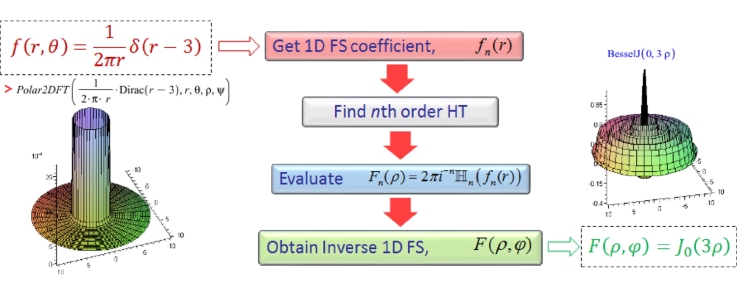

## Method details

### Requirements

• Computer algebra system (CAS) platform: Maple version 12 and higher
• Operating system: Windows XP and higher
• Additional system requirements:

If using Maple 12, minimum system requirements are 512 MB of RAM and 1 GB of hard disk space. Higher versions of Maple will require additional memory and disk space.

• Software location: Archive [1]

Name: figshare

Persistent identifier: http://dx.doi.org/10.6084/m9.figshare.1004152

Licence: CC-BY

Publisher: Natalie Baddour

Date published: 23/04/14

Language: English

• Reuse potential:

This software can be used and extended by any researchers who require the use of 2D Fourier Transforms in polar coordinates within a CAS environment. The toolbox has only been implemented in the Maple programming language.

### Introduction

This paper stems from an application in photoacoustic tomography [2], [3], [4]. Photoacoustic imaging has demonstrated great potential for the visualization of the internal structures and function of soft tissue. While performing analysis on this subject, the need arose to solve convoluted expressions exactly and analytically using integral transforms and this motivated the creation of a symbolic computer algebra system toolbox to enable easy and rapid simulations.

Prior work in symbolic computation has been in two areas; the actual algorithms in the computer algebra system, and the application of symbolic computer algebra to problems. This work endeavors to close that knowledge gap between the algorithms and the applications by creating the necessary tools (building on the algorithms) to apply in the problems. Numerical methods may be better known but some problems are still best solved using symbolic means.

### Procedure

*SCAToolbox*, the symbolic computer algebra toolbox created for our purposes, organizes a comprehensive collection of relevant, interactive computational tools into a concise, well-designed unit for easy access, usability and maintenance. It was thus, implemented as a “library” or “package” in Maple. *SCAToolbox* contains sub-packages such as the *IntegralTrans* package, containing the procedures, tables and operations necessary for computing some integral transforms, as well as several other functions (termed “procedures” in Maple), operations and tables. Hence, the toolbox has an intuitive code structure broken into four main sections – (1) creating the toolbox, (2) supporting functions, (3) integral transforms, and (4) testing and verification. The details of toolbox creation are dependent on the type of Computer Algebra Software (CAS) employed.

The path to which the toolbox and its components are stored is crucial for allowing easy access to the contents of the toolbox from any page. When the toolbox has been created, its modular nature permits modification from any file, at any time with ease. Once the toolbox is built, it can be accessed multiple times by loading its path whenever the system is restarted or a different page is opened.

Summarized below is the step-by-step process of building a toolbox:

1) Determine filename and desired path of toolbox.
2) Create the toolbox as it pertains to the chosen CAS and corresponding required syntax.
3) Initialize packages, procedures and/or tables to be included in toolbox.
4) Define desired tools within the toolbox. In our case, requisite tools include Fourier series (coefficients), Hankel transform (*n*th order), Inverse Fourier series, Shift, Scaling and Modulation rules, etc.
5) Edit those tools where necessary.
6) Save tools in sub-packages and/or toolbox as desired.
7) Save toolbox at the previously determined location.

The supporting functions are made up of operations that help manage other structures in the toolbox and are therefore critical to its completeness and effective functionality. The two types of supporting functions within this toolbox include procedures that operate on expressions (e.g., convolutions: 1D Cartesian, 2D Cartesian, angular/circular, radial, 2D Polar, series) and procedures that can manipulate other structures within the toolbox (e.g., *takeAlook*, *addToTable*, *Ekronecker* and *EdDirac* procedures). For uniformity and to minimize error, a “Bracket” convolution was implemented where the convolution type is specified as an option to a single convolution procedure. Some procedures, such as convolutions, can stand alone and so their access can be facilitated by their general placement in the toolbox without restriction to any particular sub-package.

Among the latter type of supporting functions, *takeAlook* and *addToTable* manipulate tables, a key part of our methodology. Conventional evaluation of transforms involves direct evaluation which may be time consuming and furthermore, return indeterminate results. The idea is reflective of how these kinds of problems are typically solved by hand, i.e. looking up a table of known expressions and their corresponding transforms rather than “number-crunching” each time.

A *lookup* table is used to store some basic expression- transform pairs. As a first line of action, this *lookup* table is checked when a transformation is required. The *takeAlook* procedure compares the entered expression with a previously compiled list in the first column of the *lookup* table by a process called *pattern matching*, and returns the mapping transform in the second column of the table, if a matched pattern is present. Pattern matching involves matching specific properties (arithmetic and variable types, etc.) of an expression to a fitting pattern. If the expression is not found in the table, then the *takeAlook* procedure attempts to evaluate the transform directly.

Once the transform of an unknown expression is evaluated by direct computation, the new transform pair is added to the appropriate transform *lookup* table via the *addToTable* procedure. This makes the extension of an existing table simple and efficient, preventing unnecessary computation of already known transforms and thereby saving on memory space and processing time. The output of transform procedures are written as they are entered if evaluation is unsuccessful. These table-manipulating operations are stored in the *IntegralTrans* sub- package since they are important to the inner workings of the integral transforms algorithms.

The forward and inverse *n*th order Hankel transform, and the forward and inverse 1D Fourier series are also implemented in the toolbox using *lookup* tables. The 2D Fourier transform in polar coordinates is implemented via two simpler, preceding transforms (refer to Section Additional information), rather than the less effective direct integration approach as illustrated in the example below showing the 2D Fourier transform of the shifted Dirac-delta expression (directly evaluated and using our method of applying the Hankel transform and Fourier series).

(1) $$\begin{array}{cc} DirectPolar\text{2}DFT\left(\frac{1}{2\times \pi \times r}\times Dirac(r-3),r,\theta ,\rho ,\Psi \right) & \frac{1}{2} \end{array}\frac{\int_{0}^{2\pi }e^{-{3\rho (\text{cos}(\Psi )\text{cos}(\theta )+\text{sin}(\Psi )\text{sin}(\theta ))}_{\text{d}\theta }}}{\pi }$$

(2) $$\begin{array}{cc} Polar2DFT\left(\frac{1}{2\times \pi \times r}\times \text{Dirac}(r-3),r,\theta ,\rho ,\Psi \right) & \text{BesselJ}(0,3\rho ) \end{array}$$

**Fig. 1:**
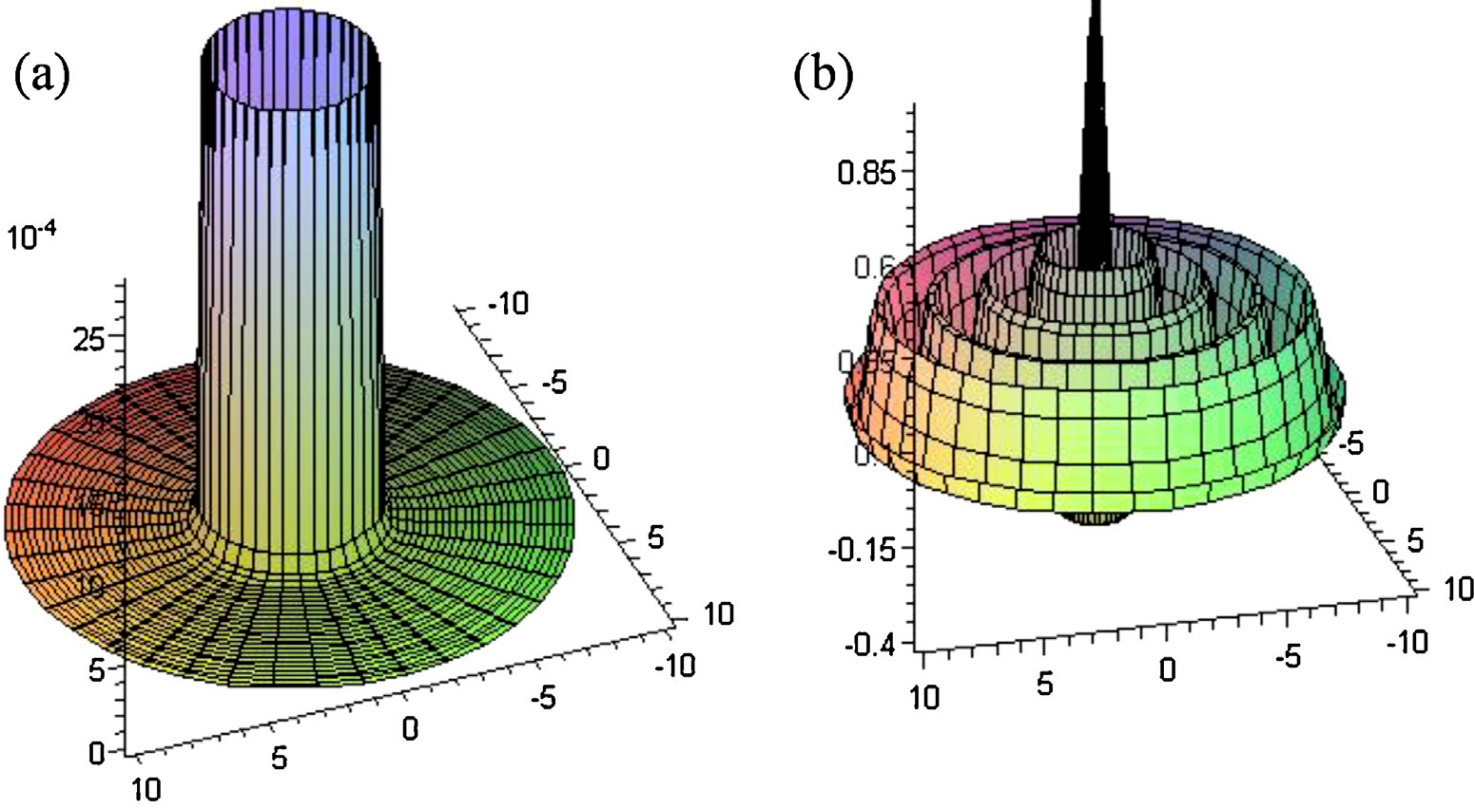
Plot of the (a) (1/2πr)δ(r−3) and its 2D polar Fourier transform, (b) $J_{0}\left(3\rho \right)$.

The direct method produces an indeterminate result whereas the indirect method gives a definite and accurate result. The plots of the shifted Dirac-delta expression and its transform are provided in Fig. 1.

## Additional information

### Theory

Computer algebra systems (CAS) provide closed-form solutions, help make direct semi-analytical, semi-numerical or symbolic-numerical methods attainable [5,6] and are useful in perturbation techniques. CAS software helps avoid a loss of accuracy during calculations and enables polynomial operations to be defined due to its ability to store variables in an *exact* form, and leave them *unassigned* (without any numerical value), respectively. Furthermore, several built-in procedures (covering general to certain specialized mathematical areas) and unique high-level programming languages exist for developing desired procedures. Symbolic computational packages therefore, make the computational implementation required in many mathematical problems, rather attractive [7].

The details of the development of the polar coordinate version of a 2D Fourier transform along with the corresponding primary rules are documented elsewhere (see [8]) but are summarized here.

The 2D Fourier transform F(ρ,ψ) of a function, f(r,θ) can be found by

1) first finding its Fourier series coefficients in the angular variable $f_{n}\left(r\right)$, given by

(3) $$f_{n}(r)=\frac{1}{2\text{π}}\int_{0}^{2\text{π}}f(r,\theta )e^{-jn\theta }\text{d}\theta ,$$

2) then finding the Fourier series coefficient of the Fourier transform, *F_n_*(*ρ*) via $F_{n}\left(\rho \right)=2\text{π}i^{-n}{ℍ}_{n}\left\{f_{n}(r)\right\}$; i.e. finding the *n*th order Hankel transform (of the spatial radial variable to the spatial frequency radial variable) of the *n*th coefficient in the Fourier series and appropriately scaling the result, and

3) finally, taking the inverse Fourier series transform (summing the series) with respect to the frequency angular variable, given by

(4) $$F\left(\vec{\omega }\right)=F\left(\rho ,\psi \right)=\sum_{n=-\infty }^{\infty }F_{n}(\rho )e^{jn\psi }.$$

### Problems encountered

The idea of building on definitions used in already-existent code to achieve a desired purpose may be attractive but it is important to understand the approaches and computational capacity of the chosen CAS platform before progressing. Some of the code may possess built-in functionalities or be proprietary and thus, unavailable for detailed analysis. Below are a few challenges encountered while developing our toolbox.

As previously mentioned, the concept of pattern matching is necessary in implementing the *lookup* tables to verify the existence of the desired function in the table. This means that all the element and variable *types* specified in the table must match those of the given input function. It is therefore important to know all the various element types in Maple.

Unfortunately, Maple defines some element types slightly differently from commonly known. For instance, Maple’s *function* type refers to a function call and not in fact to a “mathematical function”, as commonly used in most programming languages. The type *procedure* corresponds to a traditional mathematical “function,” instead. Hence, caution must be exercised when using these element types. There are also element types that consist of a group of other element types. For example, the element type ‘*algebraic*’ actually refers to *‘+’*, ‘*’, *‘^’*, *complex*, *extended numeric*, *function*, *indexed*, *name*, *series* and a few others. It is important to ensure that the element type being used is not so broad as to allow more types than necessary for a particular function, thus rendering it defective and therefore, inaccurate.

Moreover, some of the integral transforms needed in this work, such as the *n*th order Hankel transform and the 1D Fourier transform, though built into Maple, are defined differently from what is commonly known or desired and are therefore, inapplicable. Modifying such built-in functions to conform to the desired definitions could prove tedious. In our case, implementing our own function actually permitted significant intelligence and data-retention to be built in, making it easier to verify its accuracy and efficiency.

Furthermore, Maple often tries to simplify expressions within a function call as best as it can before proceeding. Any attempt at comparing the expression as entered with any property thus poses problems since what the user knows to be the starting point of the entered expression has been altered, not to a desired final result but rather to an in-between expression whose properties are unknown to the user. Since Maple does not attempt to simplify a string, entering expressions as strings can be an alternative that offers the ability to analyze and manipulate the expression as entered by the user. This also holds for operations that already exist in Maple as symbols. The symbolic representation/implementation can be bypassed by applying the quote operator. An example is in finding the 2D polar Fourier transform of a convolution. The convolution input as a string, i.e. ‘Conv’ replaces the function call *Conv*, so that the convolution remains unevaluated. This allows the standard convolution property to be matched and the convolution- multiplication rule to be applied accordingly.

It is not immediately obvious that the Maple platform itself possesses an added level of complexity which often results in inaccuracies but accommodation must be made for this. It is therefore, important to have a good understanding of how the specific CAS software works internally and to avoid over-reliance on its built-in functionalities, particularly when their details are not explicitly clear.
